# Should health insurers target prevention of cardiovascular disease?: a cost-effectiveness analysis of an individualised programme in Germany based on routine data

**DOI:** 10.1186/1472-6963-14-263

**Published:** 2014-06-17

**Authors:** Majed Aljutaili, Christian Becker, Sabine Witt, Rolf Holle, Reiner Leidl, Michael Block, Johannes Brachmann, Sigmund Silber, Kurt Bestehorn, Björn Stollenwerk

**Affiliations:** 1 Helmholtz Zentrum München, German Research Center for Environmental Health (GmbH), Institute of Health Economics and Health Care Management, Ingolstädter Landstr. 1, 85764 Neuherberg, Germany; 2 Munich Center of Health Sciences, Ludwig-Maximilians-Universität, Munich, Germany; 3 Klinik Augustinum München, Munich, Germany; 4 Klinikum Coburg, Coburg, Germany; 5 Herzzentrum an der Isar, Munich, Germany; 6 IKKF München, Institute of Clinical Pharmacology University of Dresden, Dresden, Germany

**Keywords:** Coronary heart disease, Prevention programme, Cost-effectiveness analysis, Efficiency frontier, Subgroups analysis

## Abstract

**Background:**

Cardiovascular diseases are the main cause of death worldwide, making their prevention a major health care challenge. In 2006, a German statutory health insurance company presented a novel individualised prevention programme (KardioPro), which focused on coronary heart disease (CHD) screening, risk factor assessment, early detection and secondary prevention. This study evaluates KardioPro in CHD risk subgroups, and analyses the cost-effectiveness of different individualised prevention strategies.

**Methods:**

The CHD risk subgroups were assembled based on routine data from the statutory health insurance company, making use of a quasi-beta regression model for risk prediction. The control group was selected via propensity score matching based on logistic regression and an approximate nearest neighbour approach. The main outcome was cost-effectiveness. Effectiveness was measured as event-free time, and events were defined as myocardial infarction, stroke and death. Incremental cost-effectiveness ratios comparing participants with non-participants were calculated for each subgroup. To assess the uncertainty of results, a bootstrapping approach was applied.

**Results:**

The cost-effectiveness of KardioPro in the group at high risk of CHD was €20,901 per event-free year; in the medium-risk group, €52,323 per event-free year; in the low-risk group, €186,074 per event-free year; and in the group with known CHD, €26,456 per event-free year. KardioPro was associated with a significant health gain but also a significant cost increase. However, statistical significance could not be shown for all subgroups.

**Conclusion:**

The cost-effectiveness of KardioPro differs substantially according to the group being targeted. Depending on the willingness-to-pay, it may be reasonable to only offer KardioPro to patients at high risk of further cardiovascular events. This high-risk group could be identified from routine statutory health insurance data. However, the long-term consequences of KardioPro still need to be evaluated.

## Background

Cardiovascular diseases (CVD) are the leading cause of mortality in Germany and globally [1]. They are involved in approximately 40% of all deaths before the age of 75 years in Europe [2]. Allthough CVD mortality has decreased considerably in recent decades in the United States (US) and the European Union (EU), the burden is still high [2,3]. According to the German Federal Office of Statistics, CVD caused around 350,000 deaths in 2011, and cost €37 billion in 2008 [4,5]. CVD are accountable for 17% and 10% of total healthcare expenditure in the US and the EU, respectively [3,6].

Prevention programmes include feasible and applicable measures such as the use of evidence-based medical therapies or reducing and controlling risk factors [2,3]. Reviews have shown that prevention programmes reduce CVD risks, increase the quality of life of patients and improve the health care provided [7,8]. Programmes which help control risk factors may reduce coronary heart disease (CHD) mortality by up to 50% whilst improving treatment can reduce CHD mortality by up to 40% [2].

In 2006, the German Siemens statutory health insurance company ‘Siemens-Betriebskrankenkasse’ (SBK) introduced a cardiovascular prevention programme called ‘KardioPro’. The goals of this programme are to promote prevention, early detection and guideline-based treatment of CVD. The main characteristics of KardioPro are controlling risk factors, CHD risk screening, CHD diagnosis, guideline-based therapy and an increasing number and frequency of follow-up visits with cardiologists as well as a number of prevention activities offered to the participants. In addition to the regular social health care services offered in Germany, KardioPro includes the use of computerised tomography determination of the calcium score and also angiography (CTA) for early non-invasive diagnosis of CHD. Furthermore, easy access to new treatment measures such as advanced coronary stenting technologies and the implementation of an electronic health record with easy access for the patient and the participating physicians was introduced [9].

The objective of this study was to evaluate the cost-effectiveness of KardioPro. We also aimed to quantify the cost-effectiveness of KardioPro in different CHD risk groups. On this basis, efficient strategies for targeting risk groups could be developed.

## Methods

### KardioPro

KardioPro is an individualised prevention programme, which includes screening for CHD, risk factor assessment, early detection and secondary prevention of CHD. KardioPro was first introduced in Munich and later in Coburg, Berlin, Karlsruhe, Erlangen and North Rhine-Westphalia. To enrol in KardioPro, participants needed to be at least 45 years of age. Furthermore, CHD prevalent subjects of any age were eligible for inclusion. All subjects who met the enrolment criteria received information in writing about KardioPro. Individuals in KardioPro were divided into different pathways: patients known to have CHD, suspected to have CHD and without CHD. Each pathway had a different setting depending on the individual risk or the severity of the disease. For risk assessment, the original Prospective Cardiovascular Münster (PROCAM) score was used [10]. This score defined the 10-year risk of sudden cardiac death or a definite fatal or nonfatal myocardial infarction and stratified diagnostic and therapeutic strategies. Furthermore, all KardioPro participants were categorised as either CHD prevalent subjects or subjects with high, medium or low risk, according to the PROCAM score.

The management plan for each individual patient was provided by the participating cardiologists whose task was to prescribe the optimal medical treatment for the patients and to manage the risk factors according to European guideline recommendations. Furthermore, KardioPro included recent techniques such as CT for calcium scoring (Agatston score) and/or CTA for diagnosis. In the case of necessary coronary stenting advanced types of drug-eluting stents were allowed. In addition, an electronic health record was created offering easy access for patients and facilitating communication between specialists and institutes participating in KardioPro. Patients were also sent reminders about follow-up visits.

### Subgroups and control groups

For the evaluation of KardioPro, we included participants who enrolled 2007, 2008 and 2009 and ended the observation at 31 December 2010. As the treatment paths in KardioPro differed according to risk classification, the cost-effectiveness of KardioPro was intended to be determined by risk group. However, the risk classification that was applied to KardioPro participants was not suitable for analysis, as risk classification was based on information that has only been observed for participants. The applied PROCAM score requires information about smoking behaviour, blood pressure and cholesterol level, which is not included in German sickness funds routine data [10]. Applying propensity score matching to each of the KardioPro risk groups would most likely have yielded biased control groups (selection bias), as the outcome of KardioPro depends on CHD risk. To achieve unbiased results, risk group classification needed to be consistent between KardioPro participants and controls. Furthermore, to develop efficient strategies for targeting selected risk groups (i.e. offering or advertising KardioPro to selected individuals), the risk groups needed to be defined independently from participation. Thus, for economic evaluation, new groups of patients at risk of CHD were defined based on health insurance company routine data. However, our definition of risk groups was inspired by the risk groups originally defined in KardioPro.

Firstly, we identified CHD prevalent subjects based on administrative morbidity classification. This information is included in routine data, as the presence of CHD has to be reported for each insured person within the German morbidity-based risk structure compensation scheme. The remaining subjects were divided into subjects with low, medium or high CHD risk. CHD risk scores (such as the PROCAM or the Framingham score) could not be calculated directly based on routine data. However, the PROCAM score [10], which measures the probability of CHD incidence within the next 10 years, was available for KardioPro participants. Thus, we built a regression model to predict the PROCAM score based on routine data. The routine data from CHD non-prevalent participants with a known PROCAM score at baseline of KardioPro were used as input data. As the PROCAM score is a probability and thus has a valid range from 0 to 1, we chose a quasi-beta regression model for prediction [11]. The explanatory variables used for prediction were age, sex, hypertension, obesity and diabetes, and referred to the year prior to KardioPro participation. These variables were selected as they best corresponded to prediction variables in the original PROCAM score [10]. As data for three accounting years have been combined within the regression model, we also included a random intercept for the calendar year (2007, 2008 and 2009). The concordance of the prediction model and the original PROCAM score was quantified via the Spearman’s rank correlation coefficient. The threshold values to define the low, medium and high CHD risk groups were set in such a way that the same proportions were achieved, as observed for the originally defined KardioPro risk groups (i.e. 9.72% high risk, 11.16% medium risk and 79.12% low risk among non-CHD KardioPro participants). All risk groups that we used for analysis were built based on data collected prior to enrolment in KardioPro. Thus the intervention did not affect the risk group categorisation.

Control groups were created by retrospective propensity score matching. All individuals insured by the health insurance company who did not participate in KardioPro were eligible as potential controls. However, as there are severe regional differences regarding health expenditures, only subjects from the regions where KardioPro has been offered were considered as controls. Depending on the year of enrolment, the number of KardioPro participants and potential controls were as follows: 2007: 2,953 new KardioPro participants, 131,109 potential controls, 2008 5,745 new KardioPro participants, 163,756 potential controls, 2009: 4,418 new KardioPro participants, 180,612 potential controls.

In our analysis, the propensity score was defined as the probability of the individual participating in KardioPro. The propensity score was computed by a logistic regression model based on the individual’s characteristics in the previous year (one propensity score model per calendar year). This model was selected from different logistic regression models (backward and stepwise variable selection and a full model) which were evaluated by cross-validation. Stepwise variable selection was used in the model and considered more than 140 potential explanatory variables (see Additional file 1 for a complete list of all variables included). We used the approximate nearest neighbour 1:1 without replacement approach by modifying a published macro [12]. The matching macro started to select pairs with an identical propensity score on the 10th decimal and decreased step by step until the first decimal. An additional criterion was being insured for the whole matching calendar year. Matching was done separately for each subgroup and also by year 2007, 2008 and 2009.

### Health and cost outcomes for the base case analysis

The primary health outcome was defined as event-free time and measured by counting the days until the first event (event-free days). The observation time for each matched pair started from the enrolment date of the participant in KardioPro. The observation time ended for each individual either with the first event or by censoring. Events were defined by a combined endpoint of death (all causes), myocardial infarction (MI) and stroke. MI and stroke were defined via the international classification of diseases (ICD) codes in a hospital (i.e. acute myocardial infarction (ICD-10, I21), subsequent myocardial infarction (ICD-10, I22), cerebral infarction (ICD-10, I63) and stroke (ICD-10, I64)). Censoring took place at the end of the observation period (i.e. 31 December 2010). Furthermore, the observation time was censored for both partners in a matched pair when either of them cancelled their SBK health insurance. Therefore, differences in observed time were a result of events only.

As the health outcome was calculated on a daily basis, daily health expenditures would have been desirable to calculate incremental costs. However, the routine data used for analysis reported costs for whole calendar years only, thus guiding the presentation of results.

The considered costs for each matched pair were defined as follows. For the year of enrolment, we assumed that, on account of propensity score matching, the expected health expenditures prior to enrolment did not differ systematically between the participant and the control subject. Thus, the costs for the whole calendar year were considered for each partner. In the case of censoring as a result of cancelling the SBK health insurance, which could occur on any day within the calendar year, the total costs of the particular calendar year were considered for the subject who (first) cancelled their SBK health insurance. For the matched partner however, costs were only accounted proportional to the time considered for the evaluation. When a subject died, the total reported costs of the follow-up period for both partners were considered. In cases of non-fatal events all subsequent costs for both matching partners were considered.

All costs were inflated to the year 2010 based on inflation rates provided by the German Federal Statistical Office [5]. Costs and effects were also discounted by using an annual discount rate of 5% [13]. Furthermore, costs were stratified into the following categories: hospital costs, pharmaceutical costs, physician costs and other costs. The category ‘other costs’ included, for example, physiotherapy, laboratory resources, the costs of additional services such as acupuncture and the cost of sickness benefits.

### Cost-effectiveness analysis

We assessed the cost-effectiveness of KardioPro from the statutory health insurance (SHI) perspective. The incremental cost-effectiveness ratio (ICER = (cost of intervention–cost of control)/(effect of intervention–effect of control)) was determined for KardioPro as a whole and for the individual risk groups. To illustrate which strategies are efficient, if KardioPro were to be offered to selected risk groups, we also considered combined strategies, i.e. offering KardioPro to more than one risk group simultaneously. This resulted in eight strategies overall (these are explicitly listed in Table 1). These combined strategies were graphically represented via an efficiency frontier [14,15].

**Table 1:** The base case analysis for all the strategies with ‘days until the first event (myocardial infarction, stroke or death)’ as the health outcome and cost until death

**Strategies**	**Number of pairs included in the strategy**	**Time free of event in days for participants mean (SE)**	**Time free of events in controls mean (SE)**	**Difference of days free of event per pair mean (SE)**	**Difference in time free of event in days study population (SE)**	Difference of costs per pair in €* mean (SE)	Difference in cost in €* study population (SE)	**ICER (€/year)**
KardioPro for all risk groups	13,112	629.56	629.39	3.17	41,514	674.35	8,842,036	77,742
		(1.62)	(1.65)	(0.84)	(11,070)	(133.82)	(1,754,657)	
KardioPro for CHD group	1,411	599.35	592.81	6.54	9,226	473.92	668,696	26,456
		(4.95)	(5.17)	(4.42)	(6,238)	(607.67)	(857,428)	
KardioPro for high-risk group	964	606.33	595.77	10.56	10,182	604.79	583,014	20,901
		(5.80)	(6.02)	(4.29)	(4,136)	(555.02)	(535,035)	
KardioPro for medium-risk group	1,729	604.94	599.13	5.81	10,041	832.47	1,439,334	52,323
		(4.29)	(4.40)	(2.62)	(4,537)	(425.73)	(736,090)	
KardioPro for low-risk group	9,008	641.50	640.16	1.34	12,066	682.84	6,150,992	186,074
		(1.96)	(1.98)	(0.74)	(6,694)	(138.69)	(1,249,335)	
KardioPro for CHD and high- and medium-risk group	4,104	603.34	596.17	7.18	29,558	655.71	2,690,846	33,251
		(2.83)	(2.93)	(2.13)	(8,761)	(304.32)	(1,261,563)	
KardioPro for CHD and high-risk group	2,375	602.18	594.01	8.17	19,344	527.04	1,254,061	23,678
		(3.77)	(3.93)	(3.16)	(7,501)	(425.34)	(1,000,480)	
KardioPro for high- and medium-risk group	2,693	605.44	597.93	7.51	20,241	750.96	2,016,933	36,395
		(3.45)	(3.55)	(2.28)	(6,019)	(337.36)	(910,159)	

*Only the difference in costs between intervention and control group are displayed, as the absolute values are confidential.

CHD: coronary heart diseases.

ICER: incremental cost-effectiveness ratio.

SE: standard error.

Costs were inflated to the year 2010. Both costs and effects were discounted at 5%.

### Secondary health outcome, sensitivity analyses and uncertainty assessment

Based on the number of days until death, a secondary health outcome analysis was conducted. The discount rate has been varied (at 0%, 3% and 10%) via deterministic sensitivity analysis [13]. Finally, to derive standard errors, we performed a bootstrapping approach for all the strategies by drawing 10,000 samples with replacement and calculating the ICER for each sample.

Statistical analysis was performed with the software package SAS, version 9.2.

### Ethics

The aim of this study was to evaluate the real-world health care programme KardioPro. The intervention KardioPro was offered to all persons insured by SBK as part of the health care basket provided by a German statutory health insurer, and is covered by the German Social Code §§97, 80 SGB X and §§67, 43 SGB V. KardioPro did not introduce new treatment methods, but worked by strengthening guideline-specific treatment. This type of support falls within the common responsibilities of a statutory health insurance company. Only subjects who gave written consent to participation were included in KardioPro. Evaluation of KardioPro was authorised by SBK, designed retrospectively and commissioned to an independent research group. The evaluation was based on anonymised, patient-specific routine data from SBK and was approved by the SBK data protection officer.

## Results

In the years 2007, 2008 and 2009 (the enrolment period), 13,116 individuals participated in ‘KardioPro’. For four of them, no matching control with a similar propensity score was found. After propensity score matching, 26,224 individuals (13,112 matched pairs) entered our analysis. The average age was 59.2 years for ‘KardioPro’ participants and 59.4 years for the control group. Females accounted for 45.4% of the participants and 45.8% of the control group. Differences in arterial hypertension and other co-morbidities between the two groups are shown in Table 2. For the risk level model (Table 3), the Spearman rank correlation coefficient between the originally measured PROCAM scores and the corresponding predicted risk level values was 0.74, which was highly significant (p < 0.0001).

**Table 2:** The baseline characteristics of the population of the KardioPro study

**Criteria**	**KardioPro participants**	**Control subjects**
**Female (%)**	5,950 (45.4%)	6,004 (45.8%)
**Age (years)**		
**- Mean (SD)**	59.17 (8.7)	59.42 (8.7)
**- Range**	39–88	39–94
**Arterial Hypertension (%)**	3,984 (30.4%)	4,075 (31.1%)
**Diabetes mellitus (%)**	1,537 (11.7%)	1,553 (11.8%)
**Obesity (%)**	1,665 (12.7%)	1,694 (12.9%)
**History of stroke (%)**	29 (0.2%)	17 (0.1%)
**Total**	13,112	13,112

SD: standard deviation.

**Table 3:** Quasi-beta regression with logit link function to predict the PROCAM coronary artery disease risk based on sickness funds’ routine data

**Parameter**	**Estimate**	**Standard error**	**DF**	**t statistic**	**p value**
Intercept	−1.1952	0.254	2	−4.59	0.04
Age	0.0472	0.005	1223	9.02	<0.0001
Gender (female)	−0.8587	0.103	1223	−8.31	<0.0001
Diabetes	0.0513	0.394	1223	0.12	0.91
Hypertension	0.0908	0.099	1223	0.87	0.39
Obesity	0.4230	0.124	1223	3.55	0.0004

DF: degrees of freedom.

The mean observation time in the intervention and control group was 2.39 years and 2.38 years, respectively. There were 120 (0.92%) deaths, 105 (0.80%) subjects who experienced MI and 79 (0.60%) subjects who experienced stroke in the KardioPro group, whereas in the control group there were 219 (1.67%) deaths, 114 (0.87%) subjects who experienced MI and 83 (0.63%) subjects who experienced stroke. KardioPro as a whole was associated with a statistically significant health gain (3.2 event-free days per participant, 95% confidence interval (CI) [1.7, 5.0], p = 0.0002) and a significant cost increase (€674.35 per participant, 95% CI [€438, €961], p < 0.0001). The overall cost-effectiveness of KardioPro was €77,742 (95% CI [€32,295, €260,147]) per event-free year.

Among the individual risk groups and combined strategies (Table 1), the gain of event-free days was highest in high CHD risk subjects (10.56 days SE 4.31) and lowest in low CHD risk subjects (1.34 days SE 0.74). The highest additional costs were observed within the median CHD risk subjects (832.47€ SE 420.84) and the lowest additional costs within the CHD prevalent subjects (473.92€ SE 607.50). These differences yielded a wide range of cost-effectiveness ratios, ranging from €20,901 (high CHD risk) to €186,074 (low CHD risk) per event-free year (no CIs reported, as points are scattered over multiple quadrants of the cost-effectiveness plane). Six of the eight examined strategies offering KardioPro to selected risk groups were effective in increasing event-free days (‘KardioPro for all’ p < 0.0001, ‘KardioPro for CHD prevalent subjects and medium and high CHD risk subjects’ p = 0.0006, ‘for high CHD risk and CHD prevalent subjects’ p = 0.0110, ‘for high CHD risk subjects’ p = 0.0120, ‘for medium and high CHD risk subjects’ p = 0.0004 and ‘for medium CHD risk subjects’ p = 0.0286). Four of the strategies caused significantly higher costs (KardioPro for all’ p < 0.0001, ‘KardioPro for CHD prevalent subjects and medium and high CHD risk subjects’ p = 0.0312, ‘KardioPro for medium and high CHD risk subjects’ p = 0.0276 and ‘for low CHD risk’ p < 0.0001).

**Figure 1:**
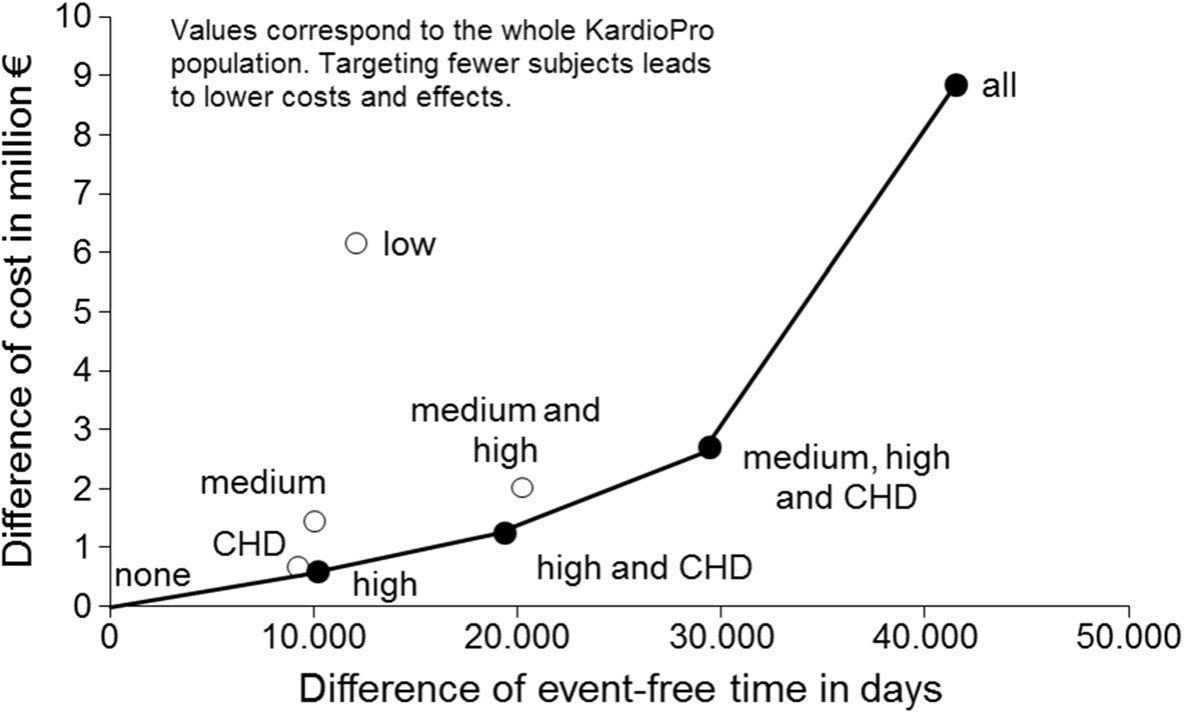
**The base case strategies with ‘days until the first event’ (myocardial infarction, stroke or death) as the health outcome and cost until death in the cost-effectiveness plane with the expansion path.** Costs were inflated to the year 2010. Both costs and effects were discounted at 5%. All: KardioPro for all risk groups. CHD: KardioPro for coronary heart disease patients group. High: KardioPro for high-risk group. Medium: KardioPro for medium-risk group. Low: KardioPro for low-risk group. High and CHD: KardioPro for high-risk and coronary heart disease patients groups. Middle, high and CHD: KardioPro for middle-risk, high-risk and coronary heart disease patients groups. Middle and high: KardioPro for middle-risk and high-risk groups. None: KardioPro for none.

**Figure 2:**
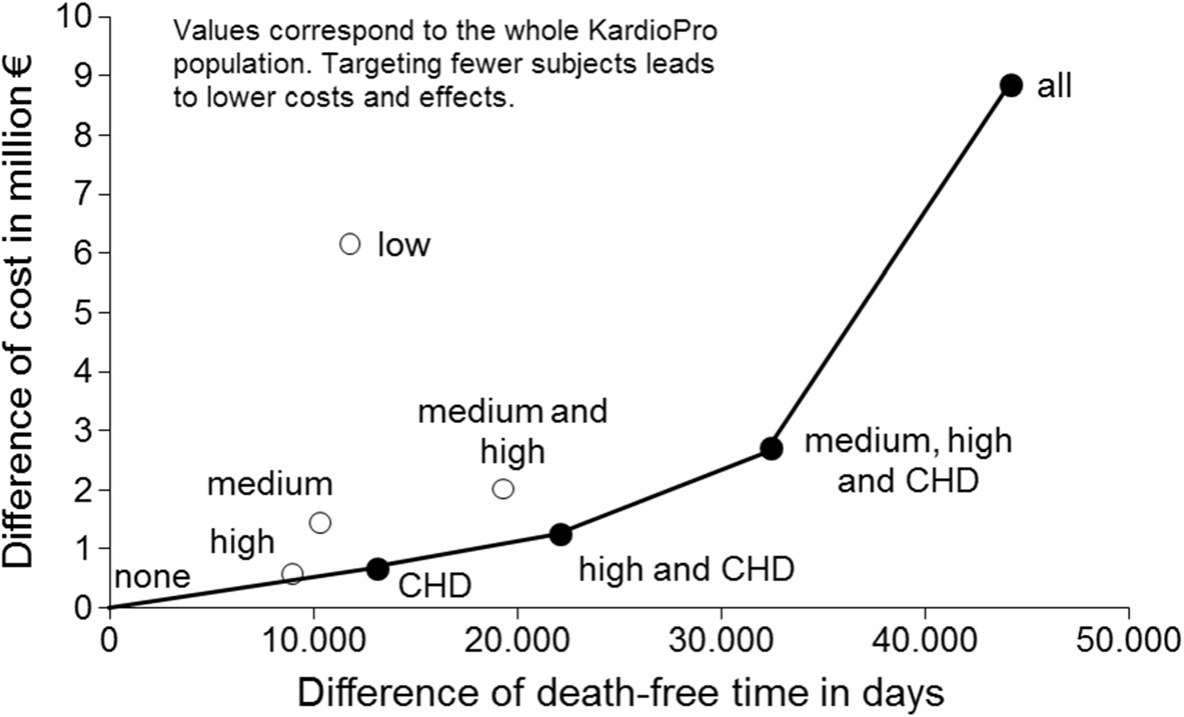
**The strategies with the secondary health outcome ‘days until death’ and cost until death in the cost-effectiveness plane with the expansion path.** Costs were inflated to the year 2010. Both costs and effects were discounted at 5%. All: KardioPro for all risk groups. CHD: KardioPro for coronary heart disease patients group. High: KardioPro for high-risk group. Medium: KardioPro for medium-risk group. Low: KardioPro for low-risk group. High and CHD: KardioPro for high-risk and coronary heart disease patients groups. Middle, high and CHD: KardioPro for middle-risk, high-risk and coronary heart disease patients groups. Middle and high: KardioPro for middle-risk and high-risk groups. None: KardioPro for none.

**Figure 3:**
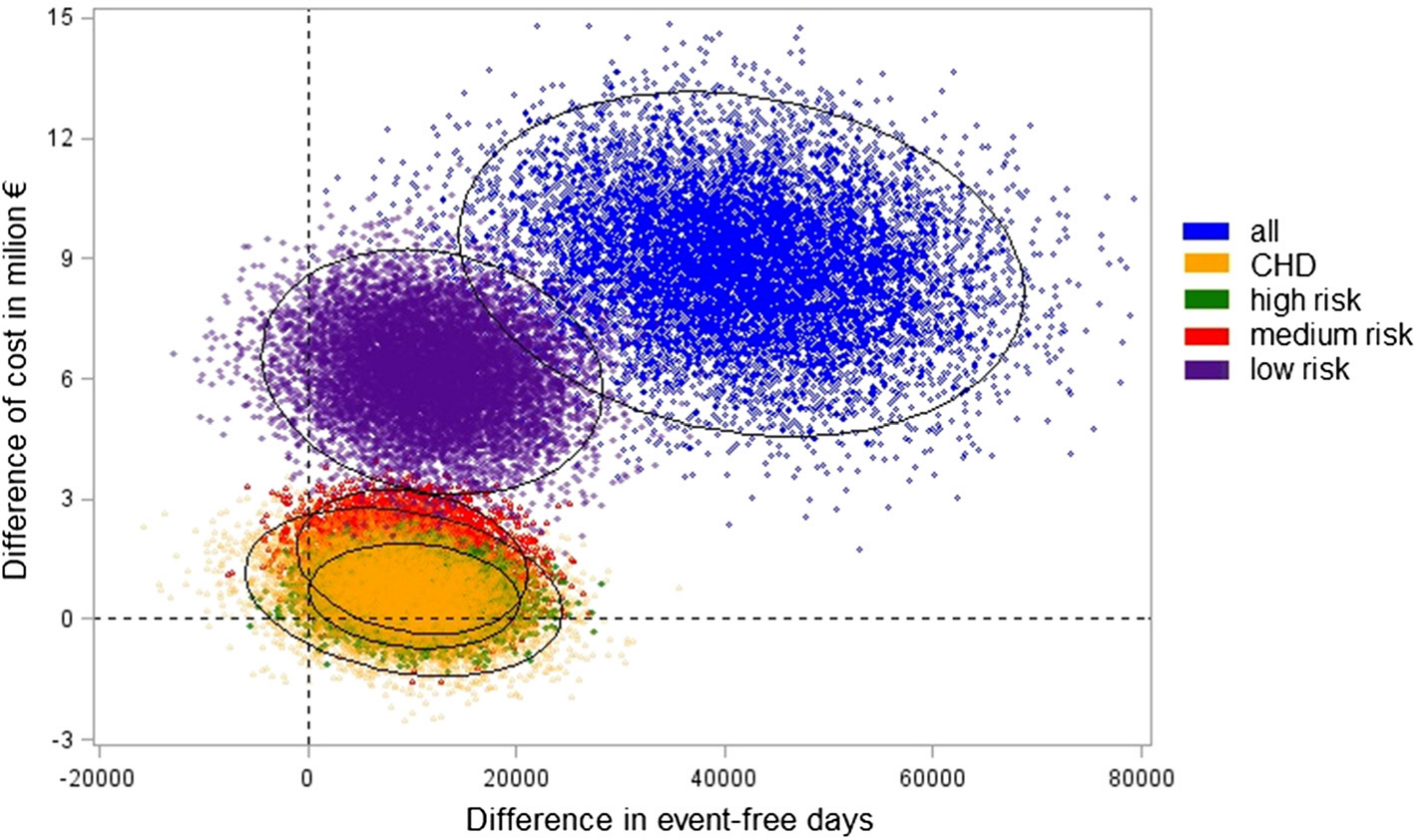
**Plot of 10,000 bootstrap samples for each strategy in the cost-effectiveness plane with 95% confidence interval ellipsoids for each.** All: KardioPro for all risk groups. CHD: KardioPro for coronary heart disease patients group. High: KardioPro for high-risk group. Medium: KardioPro for medium-risk group. Low: KardioPro for low-risk group.

Regarding the ICER, the strategy ‘KardioPro for CHD prevalent subjects’ was dominated, even though only slightly and without statistical significance, by ‘KardioPro for high risk and CHD prevalent subjects’ (see efficiency frontier, Figure 1). However, when the health outcome ‘time until death’ was used, the strategy ‘KardioPro for CHD prevalent subjects’ dominated the strategy of ‘KardioPro for high risk subjects’ (Figure 2, still only slightly and without statistical significance). A colour cost-effectiveness scatterplot based on bootstrapping for targeting the individual risk groups and KardioPro as a whole is supplied in Figure 3. A corresponding plot for all strategies that build the efficiency frontier can be found in the Additional file 2.

Among the single cost categories, physician costs contributed most to the incremental costs (Table 4). In contrast to physician costs, hospital costs were associated with cost savings due to KardioPro. The effect of the discount rate on the ICER was negligible (Additional file 3).

**Table 4:** The health expenditures of KardioPro

**Strategies**		**All health expenditures**	**Cost components**			
		**Difference in total costs**	**Difference in hospital costs**	**Difference in physician costs**	**Difference in pharmaceutical costs**	Difference in other* costs
KardioPro for all risk groups	Difference in study population in millions € (SE)	8.84	−1.63	9.14	−0.86	2.18
		(1.75)	(1.25)	(0.29)	(0.88)	(0.70)
	Mean difference per pair in € (SE)	674.35	−124.67	697.08	−65.51	253.13
		(134.42)	(96.20)	(21.68)	(66.68)	(53.74)
KardioPro for the CHD group	Difference in study population in millions € (SE)	0.67	−0.33	1.7	−0.41	−0.28
		(0.86)	(0.73)	(0.11)	(0.31)	(0.37)
	Mean difference per pair in € (SE)	473.92	−234.95	1,205.13	−287.29	38.88
		(607.50)	(513.24)	(82.18)	(217.38)	(265.70)
KardioPro for the high-risk group	Difference in study population in millions € (SE)	0.58	−0.74	0.97	0.13	0.22
		(0.54)	(0.41)	(0.08)	(0.16)	(0.24)
	Mean difference per pair in € (SE)	604.79	−768.09	1,005.87	135.68	57.04
		(554.86)	(430.80)	(81.05)	(165.36)	(252.03)
KardioPro for the medium-risk group	Difference in study population in millions € (SE)	1.44	−0.27	1.38	−0.34	0.66
		(0.73)	(0.54)	(0.09)	(0.33)	(0.25)
	Mean difference per pair in € (SE)	832.47	−153.44	799.42	−199.39	799.42
		(424.84)	(305.99)	(55.84)	(194.26)	(55.84)
KardioPro for the low-risk group	Difference in study population in millions € (SE)	6.15	−0.29	5.09	−0.24	1.59
		(1.21)	(0.75)	(0.23)	(0.74)	(0.48)
	Mean difference per pair in € (SE)	682.84	−33.01	564.81	−26.60	564.81
		(137.43)	(86.76)	(25.14)	(80.98)	(25.14)

*Other costs include costs for any other health services such as physiotherapy or laboratory services, costs for additional services such as acupuncture and also the costs of sickness benefits.

CHD: coronary heart diseases.

SE: standard error.

Costs were inflated to the year 2010 and discounted at 5%.

## Discussion

In this study, we evaluated the cost-effectiveness of the cardiovascular prevention programme KardioPro as a whole as well as in terms of strategies focused on selected subgroups of risk. As these subgroups were based on routine data, health insurance companies can easily select these risk groups for future enrolment. For rational decision makers, we also identified targeting strategies. Which of these strategies can be accepted as efficient and should be chosen, however, depends on the willingness-to-pay.

This is not the first cost-effectiveness analysis of a multi-intervention programme to prevent CVD. Field et al., for example, examined the cost-effectiveness of a range of prevention strategies, including several screening strategies and lipid lowering drugs [16]. Several disease management and health promotion bonus programmes initiated by German health insurance companies have been analysed previously [17-20]. These evaluations were also based on routine data. Yet, as subgroup analyses were not included, a special aspect about our study is that we inform decision makers on the cost-effectiveness of different individualized CHD prevention strategies.

The effectiveness and the cost-effectiveness of KardioPro differed substantially regarding the subgroups considered in our analysis. Both the effectiveness and the cost-effectiveness were most favourable in high CHD risk and CHD prevalent subjects. However, the order of cost-effectiveness among these two subgroups varied within sensitivity analyses. Nevertheless, our analysis has shown that the health effect is higher and the cost-effectiveness is more favourable in subgroups at high cardiovascular baseline risk. This association has also been observed previously, for example by Field et al. [16]. In addition it has to be kept in mind, that the effect of KardioPro was relatively small (days free of event per person between 1.34 to 10.56) during the mean observation period of 2.4 years.

The clinical effectiveness in our study was based on the combined endpoint of stroke, MI and all-cause mortality. One may wonder, why we did not refer to cardiovascular mortality, but included mortality of any cause instead. This was done for two reasons. Firstly, cause of death is not reported within health insurance company routine data. Secondly, even if we would have been able to gather the cause of death, as reported on the death certificates, this information would not have been suitable for analysis. Death certificates in Germany are known to have a high proportion of misclassification [21]. A priori we assumed that cardiovascular events, including cardiovascular mortality, were likely to be identified in KardioPro participants more frequently. To avoid bias, the ‘hard’ endpoint ‘all-cause mortality, MI and stroke’ has been applied.

Furthermore, it would have been valuable to distinguish, which costs do and which do not refer to cardiovascular interventions and procedures. However, the reported cost categories were selected a priori, were approved by the SBK data protection officer, and could not be further disaggregated in later stages of the evaluation process.

To a large extent, the health effect of KardioPro results from improvements in early diagnosis and treatment. These could be achieved through the leading role of cardiologists in the programme. Cardiologists have not only been shown to be more effective in the treatment of coronary patients, compared with general practitioners and internists [22,23], they have also been advised to precisely follow evidence-based treatment guidelines, which are currently not followed sufficiently in Germany [24]. This may explain the strong health effect in subgroups at high cardiovascular baseline risk. The increase in physician costs can also be explained by the high involvement of cardiologists. The decrease in hospital costs and costs related to medical drugs, in contrast, might have resulted from avoided cardiovascular events. However, we point out that the cost data provided for analysis was not event-specific.

We would like to stress that the time horizon of our study is relatively short. As KardioPro includes several preventive measures, the incidence of cardiovascular events is also likely to differ beyond the follow-up period. Even the costs and consequences of the events that have been observed within the follow-up period are not captured totally. Cardiovascular events are associated with long-term consequences, as they can reduce longevity and may cause or prevent future costs. Thus, it is possible that the cost-effectiveness ratios would change over time. However, there is a direct trade-off between the choice of a lifetime study horizon in the analysis of routine data and the opportunity to make timely decisions about prevention programmes including current treatment technologies.

Another limitation of our study is that it is based on observational data and not on a randomised controlled trial [25,26]. Although the propensity score matching helped to create a comparable control group, matching can only control for observed variables and thus bias can still remain [27]. For example, behavioural characteristics such as smoking, diet or exercise were not covered by the database. The same is true for some medical conditions such as gout or renal failure.

An important aspect to highlight is that we aimed to create risk groups based on routine data. The PROCAM score could not be calculated directly based on the routine data, as required variables, such as smoking behaviour, blood pressure and cholesterol level, were missing. Therefore, we created a prediction model based on the PROCAM score that was reported for participants. The PROCAM score was originally only defined for males and a certain age range [10], however, it has been applied in KardioPro as a risk indicator for women and subjects of different ages. For our economic evaluation, we were not interested in accurate estimates of the 10-year cardiovascular risk but in defining risk groups. Both the Spearman rank coefficient and our results suggest that the risk stratification was successful. However, it is conceivable that a better risk stratification could be derived from health insurance company routine data. Therefore, we recommend further research.

Regarding the health outcome, censored data such as ours are often analysed via Kaplan–Meier curves or Cox regression models. However, this was not possible given the study objective: cost-effectiveness analyses require the quantification of both costs and effects. The study design that we chose has been selected to minimise bias for the given time horizon. This has been in accordance with the analysis of censored cost data [28].

## Conclusion

In conclusion, the cost-effectiveness of KardioPro differs greatly according to the group being targeted. Subjects at high risk of CHD and with known CHD appear to be the groups that benefit most from KardioPro. Depending on the willingness-to-pay, it may be reasonable to only offer KardioPro to patients at high risk of future cardiovascular events. This high-risk group could be identified based on health insurance company routine data. However, the long-term consequences of KardioPro still need to be evaluated.

## Abbreviations

CHD: Coronary heart disease; CTA: Computerised tomography angiography; CVD: Cardiovascular disease; EU: European Union; ICD: International classification of diseases; ICER: Incremental cost-effectiveness ratio; KardioPro: Name of a cardiovascular prevention programme; PROCAM: Prospective Cardiovascular Münster Study; SAS: Name of a statistical software; SBK: German Siemens health insurance company ‘Siemens-Betriebskrankenkasse’; US: United States.

## Competing interests

Financial support for this study was provided by SBK and by Helmholtz Zentrum München, German Research Center for Environmental Health. The data was provided by SBK. The funders had no role in study design, data analysis, decision to publish, or preparation of the manuscript. However, SBK had the opportunity to comment upon the paper. MB, JB and SiS provided services for KardioPro as part of their reimbursed outpatient care.

## Authors’ contributions

BS, MA, RH and RL designed the study. MA, SW and CB performed the statistical analyses. MA, BS, RL SW, RH, MB, JB, SiS and KB interpreted the results. MA and BS drafted the manuscript. RL, SW, CB and RH reviewed and improved the manuscript. All authors revised the manuscript. MB, JB and SiS designed KardioPro (in cooperation with SBK). All the authors read and approved the final manuscript.

## Pre-publication history

The pre-publication history for this paper can be accessed here:

http://www.biomedcentral.com/1472-6963/14/263/prepub

## Supplementary Material

Additional file 1 Variables that entered the propensity score as explanatory variables within the logistic regression model. Click here for file

Additional file 2 Scatterplots on the cost effectiveness plane. Click here for file

Additional file 3 The incremental cost-effectiveness ratio for different discount rates. Click here for file
