# Supplementary material for: Should health insurers target prevention of cardiovascular disease?: a cost-effectiveness analysis of an individualised programme in Germany based on routine data
Source: BMC Health Serv Res. 2014 Jun 17;14:263. doi: 10.1186/1472-6963-14-263 (PMC4086686; doi:10.1186/1472-6963-14-263)
Supplement: Additional file 1 — Variables that entered the propensity score as explanatory variables within the logistic regression model. [file 1472-6963-14-263-S1.pdf]

Supplementary Material 1: Comparison of variables that entered the propensity score as explanatory variables within the logistic regression model

	2006	2007	2008
Age	x	x	x
Age*age	x	x	x
Age*age*age	x	x	x
Gender	x	x	x
Nationality	x	x	x
Postcode	x	x	x
Kind of insurance		x	x
Reduced earning capacity	x	x	x
Cost of medication		x	x
Cost of physician	x	x	x
Sickness benefit		x	
Cost of inpatient treatment	x	x	x
Costs for alternative medicine	x	x	x
Cost of dentist	x		x
Further costs		x	x
Number of visits to physician		x	x
Hypertension			x
Leukaemia	x		
Paresis		x	
Obesity		x	
Angiopathy	x		
Substance abuse	x	x	x
Infection by opportunistic pathogens		x	
Multiple myeloma/acute lymphocytic leukaemia			x

Selected variables for building the propensity score by logistic regression (continued)

	2006	2007	2008
Pulmonary metastasis/metastasis of digestive organ		x	x
Metastasis/Kaposi's sarcoma		x	
Metastasis in lymph node		x	x
Other severe malignant neoplasms		x	
Other malignant neoplasms			x
Other neoplasms		x	x
Diabetes with renal manifestation		x	x
Diabetes without or with unspecified complications	x	x	
Very serious metabolic disorder			x
Other severe endocrine or metabolic disorder	x	x	
Terminal liver disease		x	
Liver cirrhosis		x	x
Chronic hepatitis		x	
Chronic inflammatory bowel disease	x	x	
Oesophagitis, reflux and other diseases of the oesophagus (without ulcus and bleeding)		x	x
Inflammation/necrosis of bones/joints/muscles		x	
Rheumatoid arthritis and inflammatory connective tissue diseases	x	x	x
Spinal stenosis	x	x	x
Not postmenopausal osteoporosis	x		
Myelodysplastic syndrome and other severe haematological diseases			x
Agranulocytosis and other immune system disorders		x	
Other coagulopathy			x
Delirium and encephalopathy		x	
Dementia			x

Selected variables for building the propensity score by logistic regression (continued)

	2006	2007	2008
Schizophrenia			x
Psychosis, psychotic and dissociative disorder		x	
Depression and delusional disorders		x	
Muscular dystrophy		x	
Polyneuropathy	x	x	
Multiple sclerosis	x		
Parkinson's disease and Huntington's disease		x	
Acute pulmonary oedema and respiratory failure		x	
Heart failure		x	
Acute myocardial infarction	x	x	x
Unstabel angina and other acute ischaemic heart disease	x		x
Angina, status post myocardial infarction	x	x	x
Coronary heart disease/other chronic heart disease	x	x	x
Acquired diseases of the heart valves and rheumatic heart disease	x	x	x
Severe congenital heart defects			x
Hypertensive heart and renal disease		x	
Hypertensive heart disease		x	
Hypertension		x	
Defined arrhythmias		x	
Hemiplegia/hemiparesis			x
Not defined late effects of cerebrovascular disease		x	
Atherosclerosis		x	x
Status asthmaticus (age>17), postinflammatory pulmonary fibrosis		x	
Chronic obstructive bronchitis/emphysema (age>17), status asthmaticus (age<18)	x	x	x
Other pneumonia, emphysema, lung abscess, pleuritis		x	

Selected variables for building the propensity score by logistic regression (continued)

	2006	2007	2008
Dialysis status	x	x	x
Kidney failure			x
Nephritis		x	x
Skin ulcer (without pressure sores)		x	
Pathological fracture of the humerus, tibia or fibula		x	
Other iatrogenic complications	x	x	x
Transplantation of a major organ, receiver status			x
