# Supplementary material for: Should health insurers target prevention of cardiovascular disease?: a cost-effectiveness analysis of an individualised programme in Germany based on routine data
Source: BMC Health Serv Res. 2014 Jun 17;14:263. doi: 10.1186/1472-6963-14-263 (PMC4086686; doi:10.1186/1472-6963-14-263)
Supplement: Additional file 2 — Scatterplots on the cost effectiveness plane. [file 1472-6963-14-263-S2.pdf]

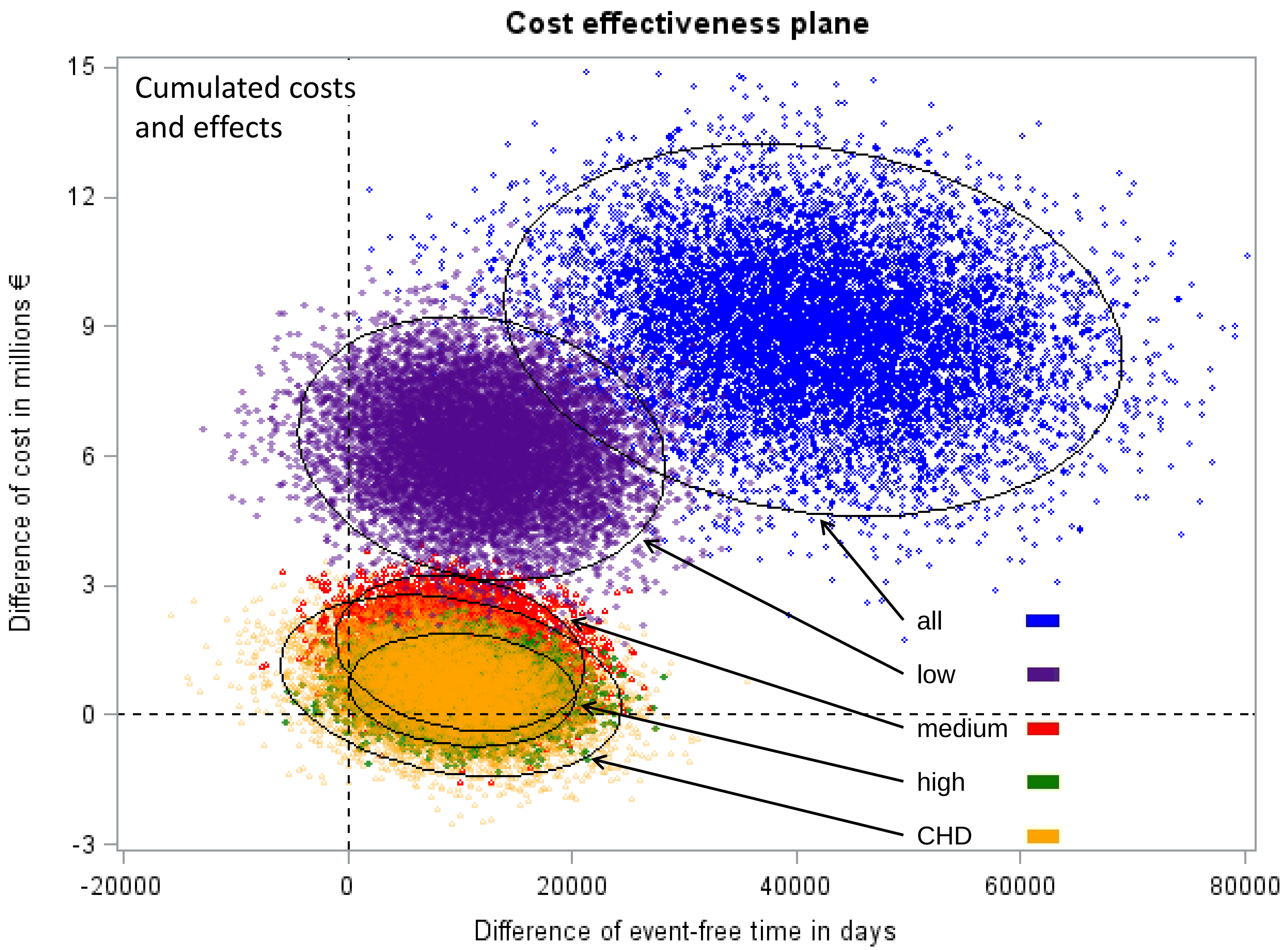

Cost effectiveness plane
Cumulated costs and effects
Difference of cost in millions €
Difference of event-free time in days
15
12
9
6
3
0
-3
-20000
0
20000
40000
60000
80000
all
low
medium
high
CHD

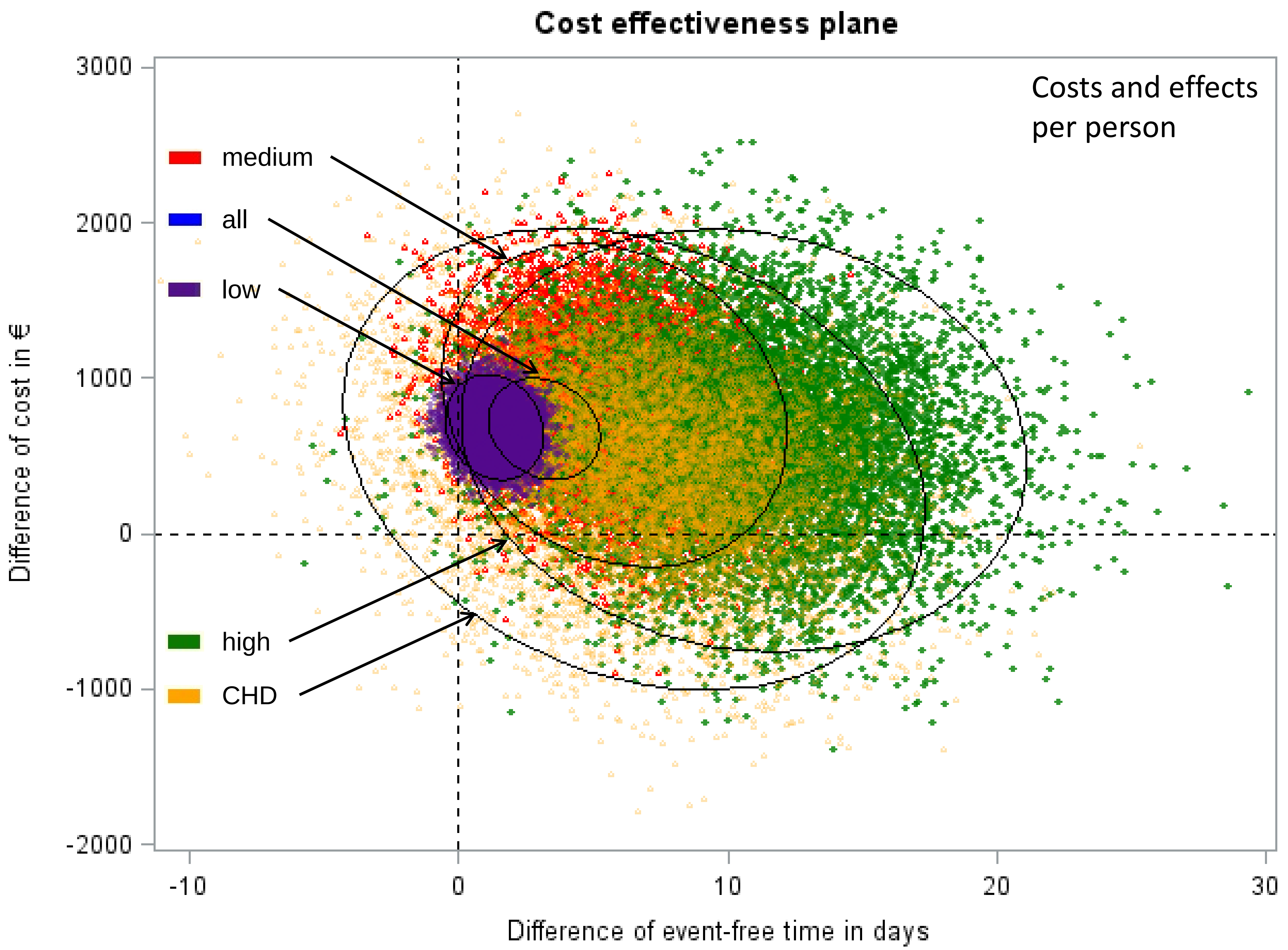
Cost effectiveness plane
Costs and effects per person
medium
all
low
high
CHD
3000
2000
1000
0
-1000
-2000
-10
0
10
20
30
Difference of cost in €
Difference of event-free time in days

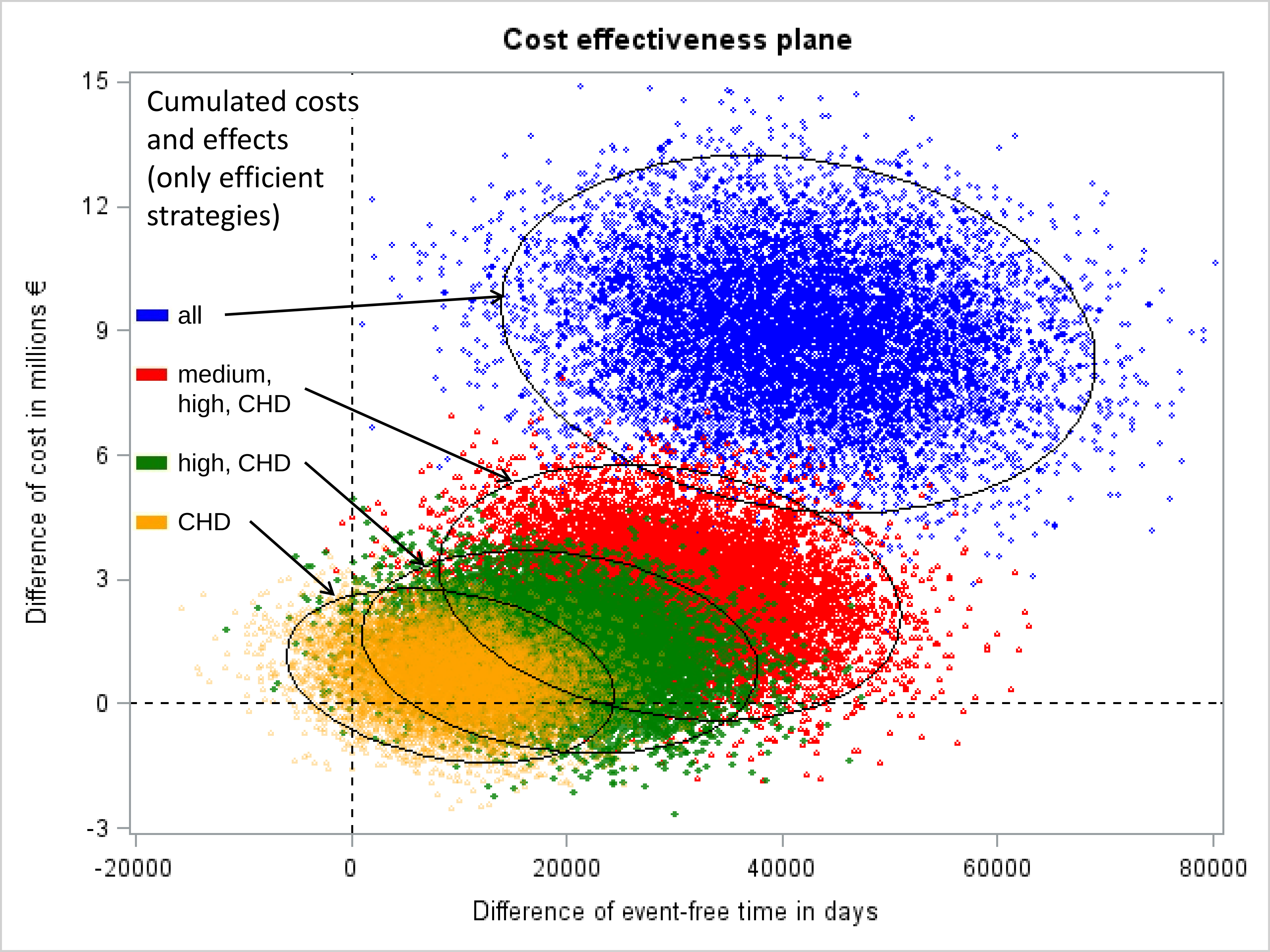
Cost effectiveness plane
Cumulated costs and effects (only efficient strategies)
all
medium, high, CHD
high, CHD
CHD
15
12
9
6
3
0
-3
Difference of cost in millions €
-20000
0
20000
40000
60000
80000
Difference of event-free time in days

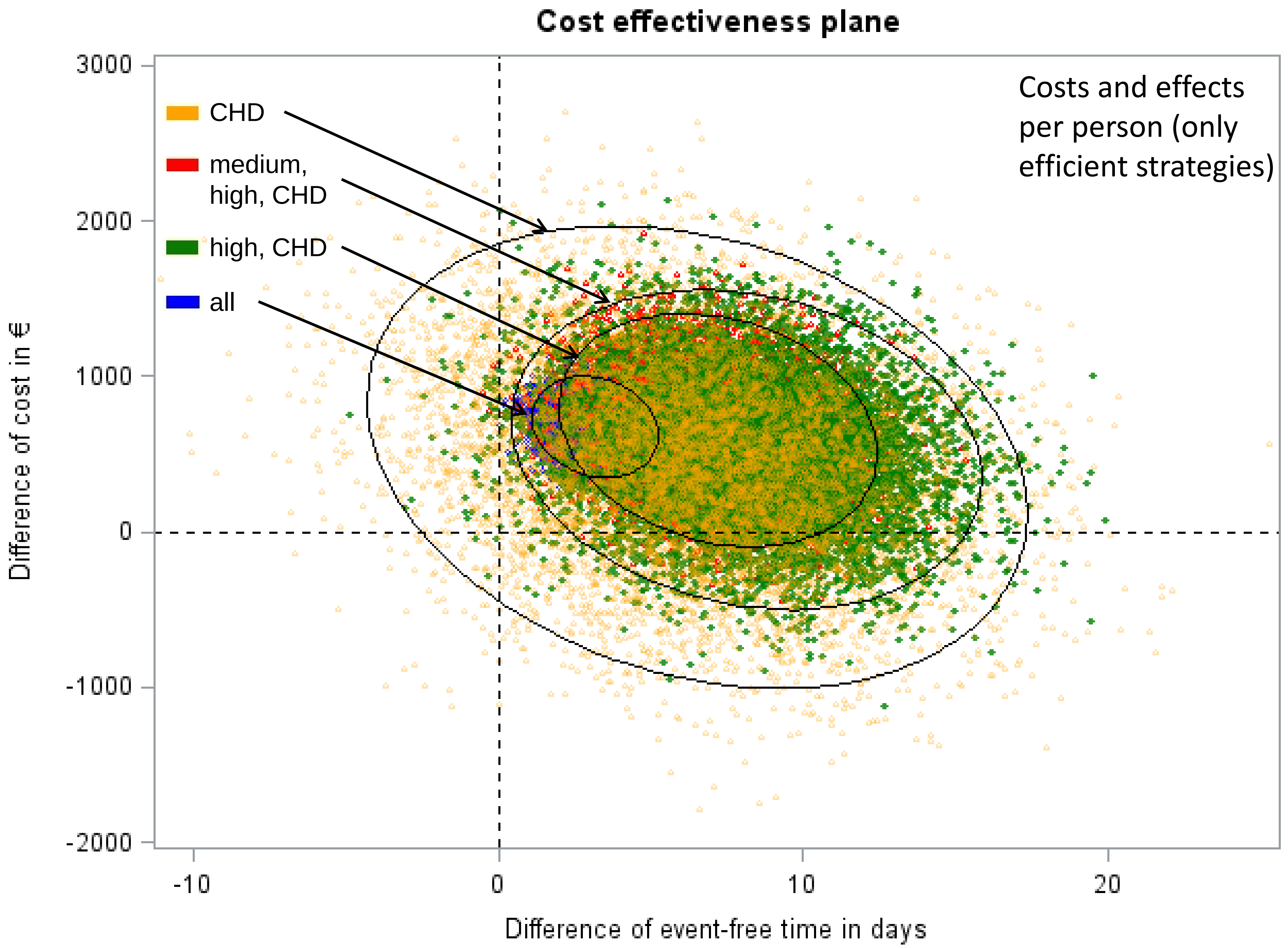

Cost effectiveness plane
Costs and effects per person (only efficient strategies)
CHD
medium, high, CHD
high, CHD
all
Difference of cost in €
Difference of event-free time in days
3000
2000
1000
0
-1000
-2000
-10
0
10
20
