# Supplementary material for: Should health insurers target prevention of cardiovascular disease?: a cost-effectiveness analysis of an individualised programme in Germany based on routine data
Source: BMC Health Serv Res. 2014 Jun 17;14:263. doi: 10.1186/1472-6963-14-263 (PMC4086686; doi:10.1186/1472-6963-14-263)
Supplement: Additional file 3 — The incremental cost-effectiveness ratio for different discount rates. [file 1472-6963-14-263-S3.pdf]

Supplementary material 3: The incremental cost-effectiveness ratio in euros per event-free year for each strategy with different discounting rates. Costs were inflated to the year 2010. *Both costs and effects were discounted at the same rate

Strategies	ICER (€/year)			
	Base case	Sensitivity analysis		
	5% Discounting*	0% Discounting*	3% Discounting*	10% Discounting*
KardioPro for all risk groups	77,742	77,654	77,749	77,727
KardioPro for the CHD group	26,456	26,411	26,448	26,470
KardioPro for the high-risk group	20,901	21,174	21,017	20,626
KardioPro for the medium-risk group	52,323	52,315	52,351	52,256
KardioPro for the low-risk group	186,074	185,007	185,749	186,846

CHD: coronary heart diseases
ICER: incremental cost-effectiveness ratio
